# A modified Delphi study to identify the features of high quality measurement plans for healthcare improvement projects

**DOI:** 10.1186/s12874-019-0886-6

**Published:** 2020-01-14

**Authors:** Thomas Woodcock, Yewande Adeleke, Christine Goeschel, Peter Pronovost, Mary Dixon-Woods

**Affiliations:** 1 NIHR ARC Northwest London, Imperial College, Reynolds Building, St. Dunstan’s Road, London, W6 8RP UK; 2 Medstar Health, Institute for Quality and Safety, 10980 Grantchester Way, Columbia, MD USA; 3 University Hospitals Management Services Center, 3605, Warrensville Center Road, Shaker Heights, OH 44122 USA; 4 THIS Institute (The Healthcare Improvement Studies Institute), University of Cambridge, Cambridge, UK

**Keywords:** Measurement, Quality improvement, Quality measurement, Delphi technique

## Abstract

**Background:**

The design and execution of measurement in quality improvement (QI) initiatives is often poor. Better guidance on “what good looks like” might help to mitigate some of the problems. We report a consensus-building process that sought to identify which features are important to include in QI measurement plans.

**Methods:**

We conducted a three-stage consensus-building approach: (1) identifying the list of features of measurement plans that were potential candidates for inclusion based on literature review and the study team’s experience; (2) a two-round modified Delphi exercise with a panel of experts to establish consensus on the importance of these features; and (3) a small in-person consensus group meeting to finalise the list of features.

**Results:**

A list of 104 candidate questions was generated. A panel of 19 experts in the Delphi reviewed these questions and produced consensus on retaining 46 questions in the first round and on a further 22 in the second round. Thematic analysis of open text responses from the panellists suggested a number of areas of debate that were explicitly considered by the consensus group. The exercise yielded 74 questions (71% of 104) on which there was consensus in five categories of measurement relating to: design, data collection and management, analysis, action, and embedding.

**Conclusions:**

This study offers a consensus-based view on the features of a good measurement plan for a QI project in healthcare. The results may be of use to QI teams, funders and evaluators, but are likely to require further development and testing to ensure feasibility and usefulness.

## Background

Prospective measurement of quality of care over time, known as *measurement for improvement*, is a defining feature of many quality improvement (QI) approaches, [1, 2] important for monitoring systems, assessing progress, and generating feedback [3]. Given its influence on the decisions and behaviours of staff, improvement teams, hospital leaders, and policy-makers, measurement quality, validity, and accuracy, analysis, and presentation are all critical. However, despite some published guidance [4, 5], the standard of measurement in QI initiatives is highly variable [6–8].

Current practice in measurement for improvement compares unfavourably with clinical trials, where high-quality measurement is recognised as a priority and accordingly is expertly led, is well-resourced, and has clear protocols for data collection. By contrast, QI teams often (albeit not always) may seem to lack capability and capacity to plan and conduct appropriate measurement: [6, 9] they often have to resort to locally designed and poorly validated measures, with data collection and analysis undertaken amid the messy realities of clinical practice [7]. These difficulties, along with the perception by some that QI measurement does not need to be rigorous, have been implicated in lack of progress and low investment in improving measurement standards [4, 8].

We propose that it is possible to mitigate some of the problems associated with measurement for improvement through meticulous and well-informed planning. The production of a written measurement plan is analogous to a research protocol: it allows QI teams to develop and communicate intentions and reach a shared understanding of how the impact of a QI initiative will be measured. This is critical, because the choices made at the planning stage (conscious or otherwise) have long-lasting implications. For example, if a team does not plan to establish baseline levels of variables, it risks not being able to evaluate the outcome of the initiative objectively.

What a QI measurement plan should include, however, has not been systematically established: little research has focused on the standards that should apply to planning for measurement for improvement. The available resources are predominantly textbooks and guides developed by organisations that support or fund QI teams. Examples include Health Quality Ontario’s Measurement Plan Tool, a checklist focusing on the data collection process [10]; NHS Scotland’s QI Hub Measure Plan and Data Collection Forms [11], developed based on a framework that is informed by Robert Lloyd’s Quality Measurement Road Map [12]; and the NHS Elect’s Measurement Checklist, which is based on its seven steps to measurement for improvement [13]. Some focus solely on a specific subset of issues, such as design of measurement. None has been developed through a formal consensus process.

In this article, we aim to address the void in guidance on planning measurement for improvement by reporting a consensus-building process to identify which features are important to include in a QI measurement plan. We do not seek to establish standards for measurement, but instead to identify the features that might benefit from standards being set, with the aim of supporting planning and review.

## Methods

Guided by a Steering Group (MDW, PP, CG), we conducted a three-stage consensus-building approach: (1) identifying the list of features of measurement plans that were potential candidates for inclusion on the basis of importance (September 2015–February 2016); (2) conduct of a modified Delphi exercise (March–May 2016); and (3) an in-person consensus group meeting to finalise the list of features (8 December 2016).

### Stage 1: Identifying the candidate features to include in the measurement plan

We generated a list of possible candidate features that could be entered into the modified Delphi study. We drew on two sources to do this.

First, we reviewed the existing literature on good (and bad) practice in measurement for improvement, including both peer-reviewed and grey literature. We started with articles recommended by members of the steering group, and then checked the reference list of these articles to identify other relevant articles. We read the articles and evaluation reports, identifying features that were mentioned either as good practice, or as mistakes or pitfalls. We added articles cited by the initial list, until this ceased to yield further candidate features. The final list of articles reviewed comprised 22 journal articles [4, 7, 8, 14–32] and 17 reports and textbooks [10–12, 33–46].

Second, we drew on the experience of the core study team (TW, YA) who have supported over 50 QI initiatives over 7 years as part of the National Institute for Health Research Collaboration for Leadership in Applied Health Research and Care (CLAHRC), Northwest London programme. We drew in particular on knowledge and experience of the challenges that teams encounter in seeking to do measurement, and of the CLAHRC’s measurement planning process, which was developed to support QI teams [47].

Consistent with the vision outlined by Berenholtz et al. [8] of producing practical tools that would be helpful to frontline staff, we decided to frame each identified candidate feature as a question that would help practitioners to review the strengths and weaknesses of plans. This process resulted in a list of candidate questions for inclusion in the modified Delphi study. For each question, we used the supporting literature to draft an explanation of what the measurement plan might include (see Table 3).

### Stage 2: Consensus-building study to select and refine the questions

A modified Delphi technique [48, 49] was used to build consensus on which questions from stage 1 were important to include as features of a QI measurement plan, using two rounds of rating and review by an expert panel over an eight-week period.

The inclusion criteria for the panel of experts invited to take part in the study were: experience of measurement planning for healthcare QI initiative(s) or specialist expertise and authority or influence in the science of improvement. Authors of this article and colleagues in the NIHR CLAHRC for Northwest London were excluded from this stage. Potential panellists were suggested by the steering group and core study team and were invited by email to take part in the study and asked to consent to participation. We asked these potential panellists to suggest others who they considered suitable to participate, who we then invited in addition, provided they met the inclusion criteria. We used Qualtrics survey software (version 1.5) to create and administer the questionnaires. We aimed to achieve a panel of 11–30 members, sample sizes in this range are typical for the Delphi method and have been shown to be effective and reliable [48, 50].

#### Delphi round 1

Round 1 of our modified Delphi used a structured questionnaire comprising the candidate questions identified in stage 1. Panellists were shown each of the candidate questions alongside the explanatory text. They were then asked to vote to keep, remove, or modify the question, or to state that they had no opinion. We used categorical response options to ensure that panellists were clear about the consequences of their votes, to make interpretation clear, and to ensure that the results were actionable in terms of establishing a final list of questions at the end of the study.

For each candidate question, panellists were given the option to provide free-text comments to support their decision, or to suggest changes to the question. We also asked panellists to give their opinion on the overall structure and completeness of the list of questions. We conducted a simple thematic analysis of free-text responses to these open-ended questions by manually searching, reviewing, defining, and naming themes [51].

Consensus was set a priori at 75% agreement with any one of the available actions (keep, remove, or modify the question), consistent with previous Delphi studies reported in the literature [52]. Responses to the round 1 questionnaire were analysed by the research team during a two-week period. We excluded responses of ‘no opinion’ from percentage agreement calculations. Any question reaching consensus to keep or remove was not fed back into the round 2 questionnaire. In cases where the panel did not reach at least 75% agreement to keep or remove a question, we examined the comments and proposed either to remove the question or to amend it to improve the framing. These questions then formed the round 2 questionnaire.

#### Delphi round 2

We asked panellists to review the aggregated agreement percentages for each question as part of the round 2 questionnaire, alongside their previous individual ratings and a summary of the panel’s comments from round 1. We then asked them to reconsider their rating using the same categories as in round 1. At the end of round 2 of the Delphi study, the analysis and feedback process was repeated.

Thus the output of the questionnaire stage was three sets of questions: a set to retain by consensus, a set to remove by consensus, and a set with no consensus to retain or to remove.

### Stage 3: In-person consensus meeting to finalise the questions

To decide what to do with the set of questions with no consensus to keep or to remove, we held an in-person meeting of the core study team and the steering group, and we also invited panellists who had completed both rounds of the Delphi study. The aim of this meeting was to finalise the structure and content of the question list. The objectives were to:

- review and resolve proposals for questions that did not reach consensus through the questionnaire rounds, drawing on panellists’ responses from stage 2
- address themes emerging from the Delphi panel’s free-text responses on the overall structure and completeness of the list of questions.

Based on the results of the Delphi study (both qualitative and quantitative), the study team proposed to retain or remove each of the remaining questions. The consensus group discussed these proposals in light of the panel voting and comments from stage 2, and accepted or rejected them, allowing the study team to finalise the question list. Any questions that reached consensus in stage 2 were not discussed in stage 3 as panel consensus was considered final.

## Results

### Stage 1: Identifying the candidate features to include in the measurement plan

We identified 104 candidate questions as potential features of measurement plans that would be relevant in reviewing strengths and weaknesses (Additional file 1: Appendix 1). We identified five high-level categories of questions: design of measurement, data collection and management, analysis, action, and embedding. These categories were further divided into ten subcategories.

### Stage 2: Consensus-building study to select and refine the questions

**Fig. 1:**
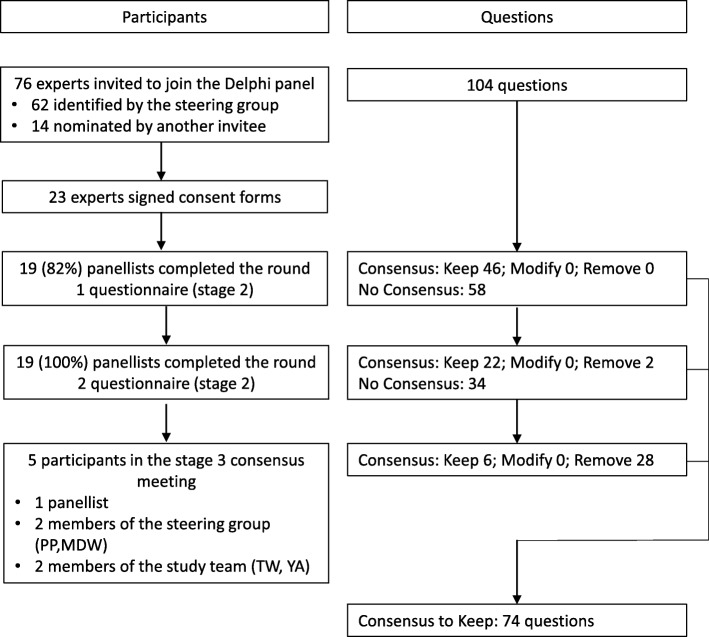
Flow of participants and questions through the study

We invited 76 experts who met the selection criteria. Figure 1 shows the flow of participants and questions through stages 2 and 3 of the study. Of the 23 panellists who consented to take part, 19 completed both rounds of the Delphi study (Table 1).

**Table 1:** Demographic characteristics of panellists in the modified Delphi study

	Panellists (*n* = 19)
**Gender**	
Male	12 (63%)
Female	7 (36%)
Country of employment	
United Kingdom	10 (53%)
United States of America	6 (32%)
Australia	3 (16%)
Role based on self-reported job titles	
Manager	8 (42%)
Academic researcher	6 (32%)
QI expert	3 (16%)
Nurse	1 (5.3%)
Doctor	1 (5.3%)
Experience in healthcare QI measurement planning	
Mean number of years	12 years

Data are number (%) unless otherwise stated. Note that some panellists held multiple roles in addition to that relating to their job title

At the end of round 1, the panel had reached consensus on keeping 46 (44%) questions. These questions were included in the final content and were not entered into round 2. The remaining 58 questions were amended based on the panel’s comments and suggestions and re-entered into round 2.

At the end of round 2, the panel had reached consensus on 24 (41%), agreeing to keep 22 and remove two questions. Thus at the end of stage 2, 70 (67%) of the 104 candidate questions met the predefined 75% agreement level: 68 to keep and two to remove. The number of questions in each subcategory reaching the specified consensus level is shown in Table 2. The panel did not reach consensus on 34 questions.

**Table 2:** Number (%) of questions in each subcategory through each stage of the study

Stage 1		Stage 2				Stage 3			
Category	Subcategory	Round 1		Round 2		Total questions	Questions removed	Questions kept	Final number of questions in each subcategory
		Total questions	Questions with consensus	Total questions	Questions with consensus				
Design of measurement	Aim	7	3 (43%)	7	5 (71%)	2	1	1	10
	Measure set	13	4 (31%)	9	4 (44%)	5	4	1	8
	Operational definition	29	16 (55%)	11	2 (18%)	9	8	1	19
Data collection and management	Data collection process	13	8 (62%)	5	0 (0%)	5	4	1	9
	Training in and embedding of consistent data collection	5	2 (40%)	3	0 (0%)	3	3	0	2
	Database design	4	1 (25%)	3	2 (67%)	1	1	0	3
	Outliers and missing data	3	0 (0%)	3	2 (67%)	1	1	0	2
Analysis	Planning the analysis	17	6 (35%)	10	3 (30%)	7	5	2	11
Action	Planning for action	4	2 (50%)	2	2 (100%)	0	0	0	4
Embedding	Planning for sustainability	9	4 (44%)	5	4 (80%) (2 to remove; 2 to keep)	1	1	0	6
Totals		104	46 (44%) (all to keep)	58	24 (41%) (22 to keep; 2 to remove)	34	28	6	74

Note that, following feedback from the panel, some questions changed subcategory after each round of the modified Delphi

### Stage 3: In-person consensus meeting to finalise the questions

For the 34 questions with no consensus at the end of stage 2, the core study team proposed to remove 27 questions and keep seven questions based on the Delphi panel’s comments. During the stage 3 in-person consensus meeting, the group (YA, TW, PP, MDW, and one Delphi panellist) agreed with removal of all 27 questions proposed as meriting removal, and with six of the seven proposals to keep questions – the seventh was deemed to be sufficiently covered by another question. Therefore in this stage six questions were kept and 28 removed.

In response to comments from the Delphi panel, the consensus group also made minor revisions to the phrasing of the six questions that were kept. This resulted in the final list of 74 questions (71% of the original 104) shown in Additional file 1: Appendix 2, along with an explanation against each question of what it means and why it is important. Two example questions are shown in Table 3.

**Table 3:** Example questions with explanations

**Question 51:** Is there a plan in place for the prospective (1) identification and (2) minimisation of missing data? [No; Yes]	
**Category 2:** Data collection and management **Subcategory 7:** Outliers and missing data	
**Explanation:** Data reviews, visual cues, and reminders can be used to identify missing data. It is important to carefully distinguish between data items that are not applicable versus missing data, and branching logic is useful for this. Methods to minimise missing data include database controls; review of the data collection tool by designated staff or an independent reviewer at the time of data entry; immediate reporting of problems to the data collection staff and project leaders – for example, if missing data are over a certain threshold, quality assurance review is needed etc. Missing data threaten the progress of QI initiatives and the validity of evaluation findings derived from them.	
**Question 61:** Is the intended frequency of feedback of the analysis to the team stated? [No; Yes]	
**Category 3:** Analysis **Subcategory 8:** Planning the analysis	
**Explanation:** Continuous communication of ongoing evaluation results to stakeholders is important for a capable improvement initiative. Updating and reporting the measures can be done on a daily, weekly, monthly, quarterly, or yearly basis. The frequency should be sufficient to see a pattern and for quicker action on the system of interest. Monthly feedback of analysis is often best suited for appropriate outcome measures, whereas weekly (or more frequent) analysis is often preferable for key process measures. However, there can also be less frequently analysed measures of interest. For instance, smoking quit rates at 1 year, or effects of lifestyle changes, may take longer to manifest.	
For QI work, it is important that at least for a small number of measures (the improvement measures), the frequency of feedback is high – ideally at least weekly. Explicitly stating the frequency of feedback at the planning stage can help to surface and deal with potential barriers to effective feedback at an early stage.	

The consensus group also discussed the themes emerging from the Delphi panel’s free-text responses on the overall structure and completeness of the list of questions, which broadly concerned whether the questions should concern methodological validity or simply transparency; the number of questions; inclusion of general project-level questions; and whether the standards that apply to research measurement should also apply to QI.

#### Methodological validity and transparency of the measurement plan

The Delphi panellists’ free-text responses highlighted some tensions about whether the measurement questions should seek solely to address the transparency of a plan (whether the methodological approach is clearly articulated) or should also seek to assess the quality of the approach to measurement. The consensus group concluded that at this stage in the development of the field, it would be difficult to add to the study’s original goal of identifying the important features of a measurement plan to seek additionally to specify the means by which quality of the methods would be assessed, especially given the complexity and context-specificity of this task. For example, decisions about which analytical methods are most appropriate for dealing with missing data depend on factors such as the extent and nature of the missing data, and the relation to other variables in the analysis. Furthermore, the decision as to which approach is optimal may involve knowledge of advanced statistical concepts that may not be readily accessible to clinical teams.

#### Number of questions

The panel raised the issue of the number of questions, noting that while the questions were comprehensive in scope, there were a lot of them taken as a whole. Given that there were already 68 questions according to the pre-defined threshold for consensus, the consensus group was careful to keep only questions deemed essential from the remaining 34 questions under consideration at the consensus meeting.

#### Inclusion of general project-level questions

Some panellists commented that some questions could be seen as not specific to measurement, but instead as pertaining to general project issues – e.g. project management or governance. The group agreed to remove any questions not specific to measurement, except those that were essential for subsequent questions to make sense. Each of these more general questions had received some votes to keep, revealing the blurred boundary between measurement and other activities undertaken by QI teams.

#### Research and QI – methodological and practical considerations

Some panellists commented that certain questions seemed more appropriate for ‘research’ rather than QI initiatives. The experts in the consensus group commented that while there are valid differences in methodology appropriate for answering different types of questions, the purpose of this study was to help bring to QI the rigor that research work benefits from. For example, in making inferences from a random sample to a fixed population versus understanding whether a change has occurred in a process over time, one would use different statistical methods. In both cases, having a clear definition of the measures used and an understanding of the quality of data against the definitions is important for the integrity of the conclusions drawn from subsequent analysis.

## Discussion

This article presents a consensus-building study aimed at identifying the important features of measurement plans for healthcare QI projects. The result is a list of 74 questions that may support QI teams in identifying the features relevant to planning and reviewing transparency and completeness of measurement planning, along with explanations for each feature. It is one of the first formal studies of this area, synthesising the cumulative learning from literature on measurement and experience in the field with the expertise of 19 leaders in the field of measurement for improvement. The findings may be of value to QI teams (for example in identifying where expert statistical or methodological advice may be necessary) and to funders, designers, and evaluators of QI programmes, though they may require further development and evaluation.

This study has a number of strengths and limitations. The initial literature search, which formed one source of candidate features for entry into stage 2 of the study, was not a systematic review. It is therefore possible that some potential candidate features present in the literature were missed, and that some candidate features used in the study are not evidenced in the literature. Use of the modified Delphi technique in Stage 2 offered a number of advantages, preserving the anonymity of panellists and allowing unrestricted expression of opinions, and thus helping to reduce the influence of dominant personalities and the effect of panellists’ status on results [49]. It allowed the panel to choose freely whether to keep, modify, or remove questions. It also permitted coverage of the full range of QI initiatives, rather than, for example, focusing on those based in hospitals or particular health conditions. However, the long list of questions that emerged – over 70 – offers insight into the complexity of measurement as an endeavour in QI work. Such complexity is a theme emerging in the study of QI more generally [53], but the number of questions may be a risk to feasibility for general use and, if unattainable, may risk alienating or demoralising teams. Face-to-face discussions in Stage 3 enabled decisions to be made where no consensus could be reached using the remote survey in stage 2, but may have been vulnerable to typical group norms and effects. The study has helped to identify requisite features of a good plan and whether they are articulated transparently, but has not addressed the issue of quality of methods. This may be a focus of future work.

Further research is also needed to understand the relative importance of the questions identified through this study, to allow prioritisation of resources in planning improvement, and to convert the long list of questions we have identified into a practically useful guide for QI teams. Such work might focus on systematic approaches to help teams develop measurement plans that are scientifically valid, practically feasible, and promote successful improvement. This may require, for example, presenting the study findings in a user-friendly format suitable for QI teams, perhaps through development of an interactive guidance and support tool to facilitate adoption of the findings of this study. Such a tool could also provide useful data on which areas of measurement planning are particularly challenging for QI teams, and therefore where systematic support might best be aimed. A prototype tool has been developed [54], and the authors plan to report its development in a subsequent article.

## Conclusions

Existing checklists and templates to support measurement planning are not comprehensive and none has been developed through a formal expert consensus technique. QI teams may use the results of our study proactively to highlight areas where they need to seek additional expertise, or to develop their plans further. Further work may be needed to refine and test the tool to ensure feasibility and usefulness, and to ensure that QI teams are appropriately supported in developing and reviewing measurement plans.

## Supplementary information

**Additional file 1:** **Appendix 1.** Table of candidate questions for measurement plan review. **Appendix 2.** Consensus results: table of measurement plan review questions.

## Data Availability

The anonymised datasets analysed in this study are available from the corresponding author on reasonable request.

## Abbreviations

CLAHRC: Collaboration for Leadership in Applied Health Research and Care
QI: Quality Improvement
